# X-ray excited luminescence spectroscopy and imaging with NaGdF_4_:Eu and Tb†

**DOI:** 10.1039/d1ra05451a

**Published:** 2021-09-24

**Authors:** Meenakshi Ranasinghe, Md. Arifuzzaman, Apeksha C. Rajamanthrilage, W. R. Willoughby, Ashley Dickey, Colin McMillen, Joseph W. Kolis, Mark Bolding, Jeffrey N. Anker

**Affiliations:** Department of Chemistry, Center for Optical Materials Engineering and Technology (COMSET), Clemson University Clemson SC USA janker@clemson.edu; Department of Radiology, University of Alabama at Birmingham School of Medicine Birmingham AL USA

## Abstract

X-ray excited optical luminescence from nanophosphors can be used to selectively generate light in tissue for imaging and stimulating light-responsive materials and cells. Herein, we synthesized X-ray scintillating NaGdF_4_:Eu and Tb nanophosphors *via* co-precipitate and hydrothermal methods, encapsulated with silica, functionalized with biotin, and characterized by X-ray excited optical luminescence spectroscopy and imaging. The nanophosphors synthesized by co-precipitate method were ∼90 and ∼106 nm in diameter, respectively, with hydrothermally synthesized particles showing the highest luminescence intensity. More importantly, we investigated the effect of thermal annealing/calcination on the X-ray excited luminescence spectra and intensity. At above 1000 °C, the luminescence intensity increased, but particles fused together. Coating with a 15 nm thick silica shell prevented particle fusion and enabled silane-based chemical functionalization, although luminescence decreased largely due to the increased mass of non-luminescent material. We observed an increase in luminesce intensity with temperature until at 400 °C. At above 600 °C, NaGdF_4_:Eu@SiO_2_ converts to NaGd_9_Si_6_O_26_:Eu, an X-ray scintillator brighter than annealed NPs at 400 °C and dimmer than NPs synthesized using the hydrothermal method. The particles generate light through tissue and can be selectively excited using a focused X-ray source for imaging and light generation applications. The particles also act as MRI contrast agents for multi-modal localization.

## Abstract 2

We synthesized and characterized Eu and Tb doped NaGdF_4_ nanophosphors which generate visible light when excited by a focused X-ray beam. High resolution images were acquired through tissue by measuring light intensity *vs.* X-ray beam position.

## Graphical abstract

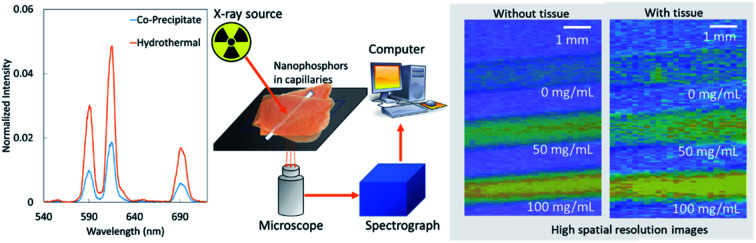

## Introduction

Light can be a powerful tool for studying biochemistry in cells and tissue and for stimulating responses from genetically engineered light-sensitive neurons (optogenetics) or photo-released drugs. The challenge for delivering or retrieving light from deep tissue is that scattering in the tissue prevents light from travelling ballistically, and the point spread function is typically >1 cm through 1 cm of tissue, also the light is attenuated by the tissue.^1,2^ One approach that circumvents these problems is to use selectively generate light using focused X-ray beams to generate visible luminescence from scintillators implanted or injected in the tissue. Since X-rays are far less scattered than visible and near-infrared light, the X-ray beam focus can be maintained through the tissue and generate visible light locally after absorption by scintillator particles. Scintillators are luminescent materials that absorb high-energy radiation (γ- and X-ray photons, high energy ions, neutrons, or other subatomic particles) and emit UV, visible and/or NIR light.^1,2^ These materials are widely used in radiation detection^3^ and imaging applications,^4–6^ and occasionally in external power-free light generation applications (*e.g.*, old luminescent watches, emergency lighting and gun sites).^7^ Recently, researchers have been developing scintillator nanophosphors for improved X-ray luminescence imaging,^8–10^ radiation imaging,^9,11,12^ and as a potential light source for photochemistry and photobiology (*e.g.*, X-ray excited optogenetics^13^).

Rare-earth doped materials (NaGdF_4_:Eu^14,15^ and Tb,^16^ GdO_2_S_2_:Eu and Tb,^10^ YAG:Nd, LuAG:Tm, Lu_2_SiO_5_:Ce^17^) have interesting properties including narrow and distinct spectral emission peaks, large optical penetration depth, negligible autofluorescence background, photochemical stability and low toxicity.^6^ Among them, rare-earth fluorides (NaGdF_4_) are considered excellent host matrices for luminescent rare earth elements (*e.g.*, Eu and Tb). They usually have high refractive indices and low phonon energies, which cause a low probability of nonradiative decay and higher luminescent quantum yields compared to oxide hosts and other inorganic matrices.^18^

Herein, we synthesize 100 nm diameter NaGdF_4_:Eu and NaGdF_4_:Tb nanophosphors for X-ray excited luminescence. This diameter is in a good range for long circulation as nanoparticles smaller than ∼5.5 nm undergo relatively fast blood clearance by renal filtration, nanoparticles smaller than ∼70 nm filter through fenestrae of hepatic sinusoids,^19–21^ while nanoparticles between 200–500 nm are endocytosed by phagocytic or non-phagocytic cells and nanoparticles larger than 500 nm are likely to remove form cells by phagocytosis.^19,22^ Thus nanoparticles with 100 nm diameter have shown the minimum effect on blood clearance mechanisms (ex. renal, liver filtration) and prolonged circulation time.^20,21^ These intermediate size nanoparticles tend to accumulate in perivascular spaces of permeable tissues (ex. liver and spleen, tumour, site of inflammation and angiogenesis)^19,20^ allowing bicomponent imaging.

We also compared X-ray excited optical luminescence (XEOL) spectra from hydrothermal and co-precipitation synthesis methods and the effect of thermal annealing. Nanoparticle annealing at high temperature is a widespread method in upconversion nanophosphors to enhance luminescence^10,23,24^ by reducing crystal defect, increasing crystal domain size and removing trapped water and carbon dioxide. However, enhancing luminescence of X-ray excited nanophosphors using annealing techniques is still under investigation. Here, we synthesized both NaGdF_4_:Tb and NaGdF_4_:Eu, which have distinct spectra. The nanophosphors were functionalized with biotin and attach to streptavidin *in vitro*. They could be selectively excited in a solution using a focused X-ray source to obtain XEOL spectroscopy and to image through tissue; here we focused on the NaGdF_4_:Eu because their red emission has deeper penetration through tissue than the largely green emitting NaGdF_4_:Tb.^25,26^ Additionally, the particles serve as *T*_1_ and *T*_2_ MRI contrast agents which would be useful for multi-modal imaging applications.

## Results and discussion

We synthesized NaGdF_4_:Eu and NaGdF_4_:Tb nanophosphors *via* co-precipitation and hydrothermal methods and studied the effect of dopant concentration on their optical spectra and crystal diffraction patterns. Next, we encapsulated the nanophosphors in silica to prevent thermal sintering and studied the effect of thermal treatment. We functionalized the nanophosphors with biotin and showed that we could excite colloidal suspensions of particles with a focused X-ray beam and image the luminescence through tissue. Finally, we explored the possibility of multimodal imaging based on spectrally distinct features of the nanophosphors as well as MRI contrast.

### NaGdF_4_:Eu and NaGdF_4_:Tb nanoparticle synthesis

In the NaGdF_4_ nanophosphor synthesis, citrate acts as a complexing and dispersing agent that forms a La^3+^–Cit^3−^ complex and disperse in deionized (DI) water. Upon addition of excess NaF, La^3+^ released from the complex reacts with Na^+^ and F^−^ to form NaGdF_4_ nuclei. After the formation of NaGdF_4_:Eu and Tb, the citrate serves as a surfactant which controls nanoparticle growth and prevents aggregation.^15,27^

#### Co-precipitate synthesis method

**Fig. 1:**
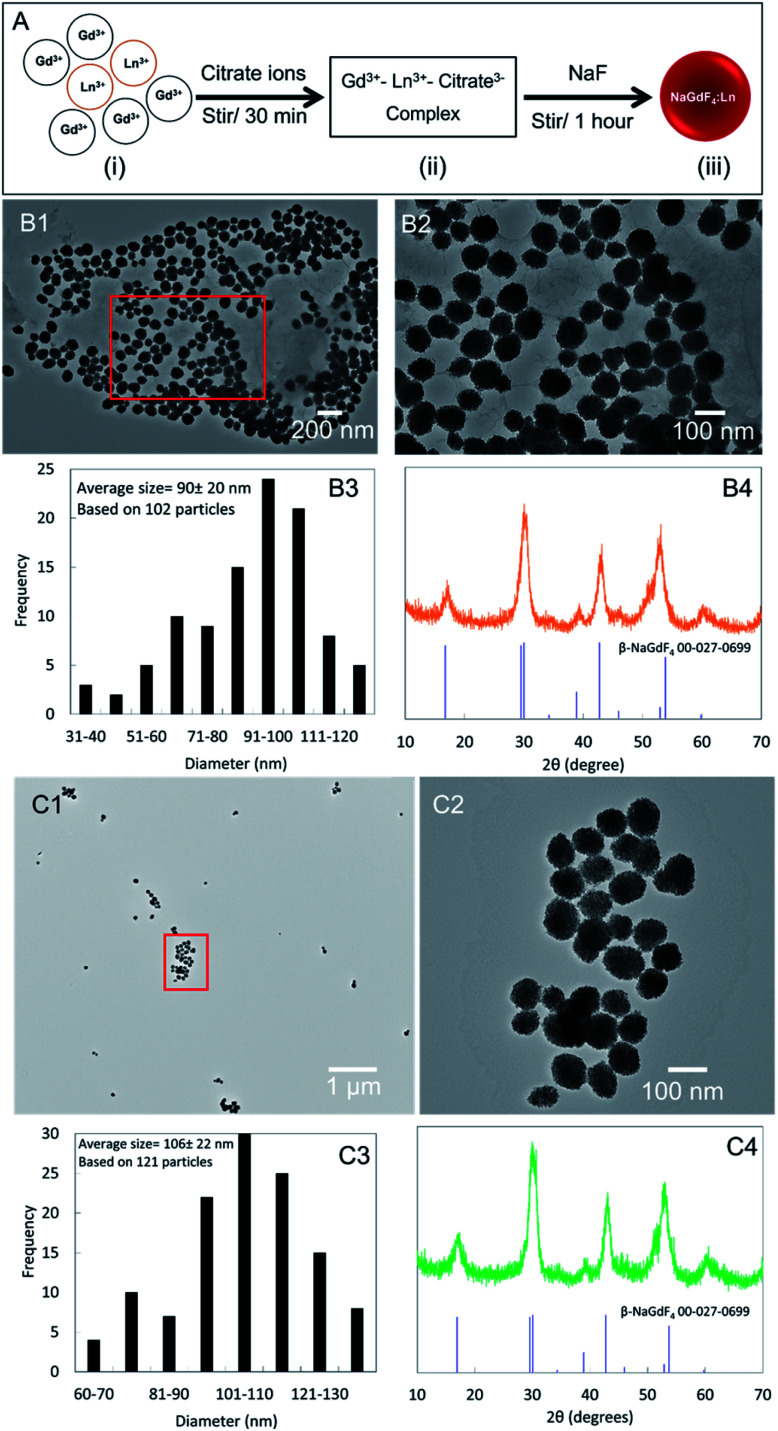
(A) Schematic illustration of nanophosphors synthesis according to co-precipitate method. (i) Mixture of Gd(NO_3_)_3_ and Eu(NO_3_)_3_ or Tb(NO_3_)_3_, (ii) Ln^3+^–citrate^3−^ complex appears as a clear solution, (iii) formation of NaGdF_4_:Ln^3+^ nanophosphors. Zoom out TEM images of (B1) NaGdF_4_:Eu and (C1) NaGdF_4_:Tb nanophosphors. Zoom in TEM images of (B2) NaGdF_4_:Eu and (C2) NaGdF_4_:Tb nanophosphors. Histograms of the average diameter of (B3) NaGdF_4_:Eu and (C3) NaGdF_4_:Tb. Powder XRD pattern compared to PDF cards 27-0699 of (B4) NaGdF_4_:Eu and (C4) NaGdF_4_:Tb.

The co-precipitate synthesis process is presented in Fig. 1A. The nanophosphors synthesized using the co-precipitate method at room temperature were approximately spherical in shape (Fig. 1B1 and C1). The Eu-doped and Tb-doped nanophosphors respective had average diameters of ∼90 nm (Fig. 1A2) and 106 nm (Fig. 1C2). The powder X-ray diffraction (powder-XRD) peaks displayed the presence of hexagonal (β-phase) NaGdF_4_ (Fig. 1B3 and C3) indexed to the standard data (JCPDS 00-027-0699), with relatively large peak with indicating small crystal domain sizes (∼55 Å NaGdF_4_:Eu and ∼53 Å NaGdF_4_:Tb according to the Scherrer equation based on peak at 16.8 degrees). TEM images and powder-XRD patterns of Eu- and Tb-dopped NaGdF_4_ (0.1, 1, 15, 20 and 100%) are included in ESI S1.† The synthesized nanophosphors show powder-XRD peak shift compared to standard peak positions (ex. peaks at around 53°). This observation is common in most powder-XRD patterns due to changes in stoichiometric composition by doping (size and amount of dopant), microstructure parameters (crystallite size and lattice strain).^28^ X-ray luminescence nanophosphors with low emission intensity are used in X-ray luminescence computer tomography (XLCT) and focused X-ray luminescence tomography (FXLT).^11^

**Fig. 2:**
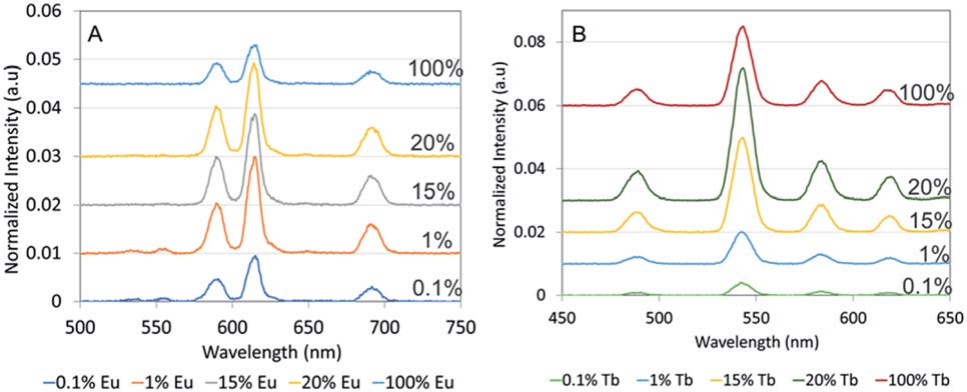
X-ray excited optical luminescence (XEOL) of (A) NaGdF_4_:Eu and (B) NaGdF_4_:Tb nanophosphors. Eu and Tb dopant fraction (mol% added to reagents *vs.* Gd) of NaGdF_4_ nanophosphors varied from 0.1% to 100%. X-ray luminescence intensities of nanophosphors were normalized to the 620 nm peak intensity from commercial GOS:Eu and Tb microphosphors. Spectra have been vertically displaced for ease of comparison.

The optical properties of rare-earth doped nanophosphors depend upon the doping ions and crystalline matrix. Fig. 2 shows the X-ray excited optical luminescence spectrum of NaGdF_4_:Eu. There are three characteristic peaks in the visible range of the electromagnetic spectrum, at around 589 nm, 615 nm and 691 nm that corresponding to ^5^D_0_ → ^7^F_1_, ^5^D_0_ → ^7^F_2_, ^5^D_0_ → ^7^F_4_ transitions.^10,14^ Tb doped NaGdF_4_ have four characteristic peaks in the visible range, at around 489 nm, 543 nm, 585 nm and 620 nm that corresponding to ^5^D_4_ → ^7^F_6_, ^5^D_4_ → ^7^F_5_, ^5^D_4_ → ^7^F_4_, ^5^D_4_ → ^7^F_3_ transitions.^10,29^ Since the luminescence intensity depends upon dopant concentration,^30,31^ we varied the amount of Eu and Tb compared to Gd^3+^ in the reaction mixture. Fig. 2A and B show the luminescence spectra for Eu- and Tb-doped nanophosphors, respectively. The dopant:Gd molar concentration ratios in the NaGdF_4_ synthesis reaction were 0.1% (bottom), 1%, 15%, 20% and 100% (top). A base value has been added to each spectrum to avoid spectral overlapping. At low and high molar ratios of both Eu and Tb doped NaGdF_4_ nanophosphors showed low luminescence intensity due to lack of luminescence centres and self-quenching (cross relaxation between two neighbouring Eu^3+^),^30^ respectively. Intermediate concentrations, 1%, 15% and 20% in Eu-doped NaGdF_4_ showed the highest although similar luminescence intensities. In Tb-doped NaGdF_4_ 20% showed the highest intensity.

#### Hydrothermal synthesis method

**Fig. 3:**
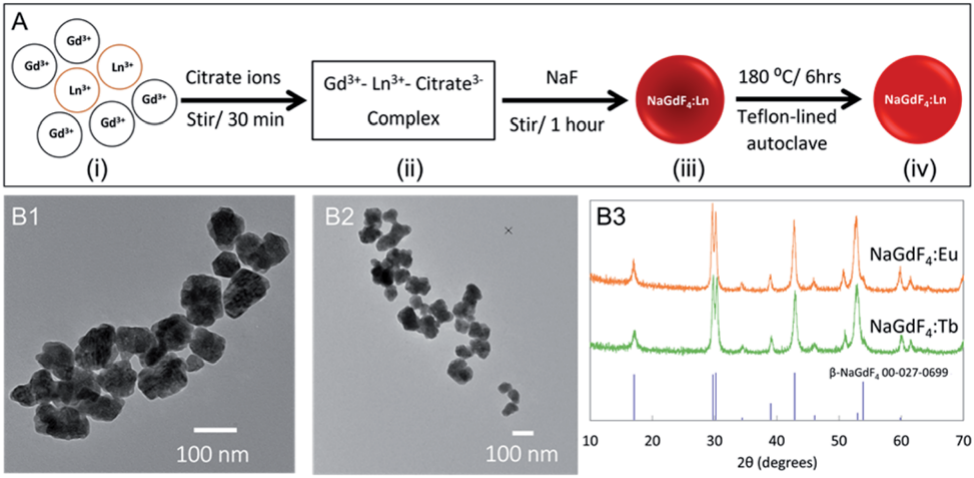
(A) Schematic illustration of nanophosphors synthesis according to co-precipitate method. (i) Mixture of Gd(NO_3_)_3_ and Eu(NO_3_)_3_ or Tb(NO_3_)_3_, (ii) Ln^3+^–citrate^3−^ complex appears as a clear solution, (iii) formation of NaGdF_4_:Ln^3+^ nanophosphors, (iv) hydrothermally treated NaGdF_4_:Ln^3+^ nanophosphors. TEM images of hydrothermally treated (B1) NaGdF_4_:Eu and (B2) NaGdF_4_:Tb nanophosphors. (B3) Powder XRD pattern compared to PDF cards 27-0699 of NaGdF_4_:Eu and NaGdF_4_:Tb.

The hydrothermal synthesis process is presented in Fig. 3A. The crystallinity of inorganic crystals plays an important role in X-ray luminescence intensity where large crystal domain size results in high luminescence intensity.^10^ During the hydrothermal process, nanophosphors recrystallize to rearrange the crystal structure. The nanophosphors synthesized using the co-precipitate method at room temperature were hydrothermally treated to increase the crystallinity and thereby, enhance the X-ray luminescence intensity. The hydrothermally treated nanophosphors were irregularly shaped (Fig. 3B1 and B2) as nanophosphors are starting to aggregate and fuse at high temperature. However, narrow peaks in the powder XRD pattern (Fig. 3B3) confirmed the increased crystallinity compared to nanophosphors synthesized at room temperature. (∼179 Å NaGdF_4_:Eu and ∼128 Å NaGdF_4_:Tb according to the Scherrer equation based on peak at 17 degrees). TEM images and powder-XRD patterns of 0.1–100% Eu- and Tb-dopped NaGdF_4_ are included in ESI S2.†

**Fig. 4:**
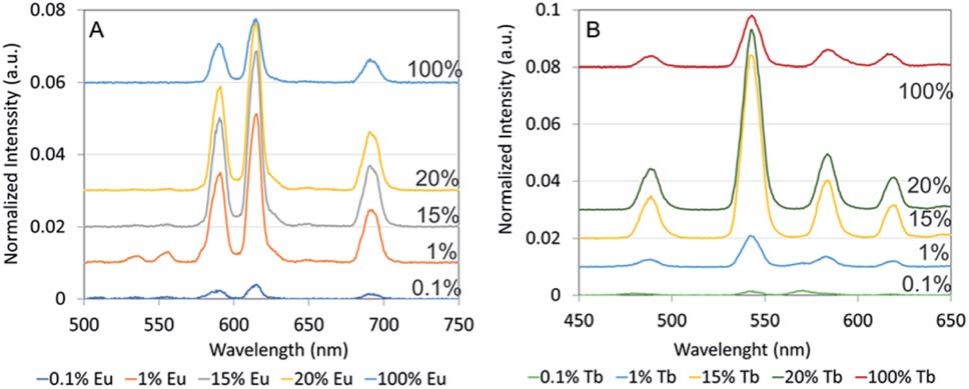
XEOL of hydrothermally treated (A) NaGdF_4_:Eu and (B) NaGdF_4_:Tb nanophosphor. Mol fraction of Eu and Tb dopant *vs.* Gd in regent mixture varied from 0.1% to 100%. X-ray luminescence intensities of nanophosphors were normalized to the height of 620 nm peak of commercial GOS:Eu and Tb microparticles. Spectra have been vertically displaced by adding a baseline for ease of comparison.

Hydrothermally treated nanophosphors showed enhanced X-ray luminescence intensity by a factor of 2–2.5 compared to nanophosphors synthesized at room temperature. 15% Eu doped NaGdF_4_ showed the highest intensity however, 1% and 20% Eu doped NaGdF_4_ showed high values close to the highest intensity (Fig. 4A). The X-ray luminescence intensity of 15% Tb doped NaGdF_4_ had the highest intensity and 20% Tb doped NaGdF_4_ showed an intensity close to the highest value (Fig. 4B)

### Inductive coupled plasma-optical emission spectroscopy (ICP-OES) metal analysis

ICP-OES analysis was performed for Gd, Eu and Tb to determine the Eu/Gd and Tb/Gd ratios of the synthesized nanophosphors, which determine X-ray luminescence intensity. All four types of samples (NaGdF_4_:Eu and NaGdF_4_:Tb synthesized by co-precipitate and hydrothermal methods) show increased percent metal ratio as the fraction of dopant added increases. The amount of dopant in the final systems was directly proportional to amount added to the reagent mix, with a slope of 1.2 for both the coprecipitated and hydrothermal NaGdF_4_:Eu (*i.e.*, nanophosphors incorporated 20% more Eu than was added to the reagent mixture) and 1.3 for the NaGdF_4_:Tb (*i.e.*, nanophosphors incorporated 30% more Eu than was added to the reagent mixture), suggesting increases in thermodynamic stability or kinetic reaction rates during synthesis.

### Silica-coating NaGdF_4_:Eu and Tb nanophosphors

**Fig. 5:**
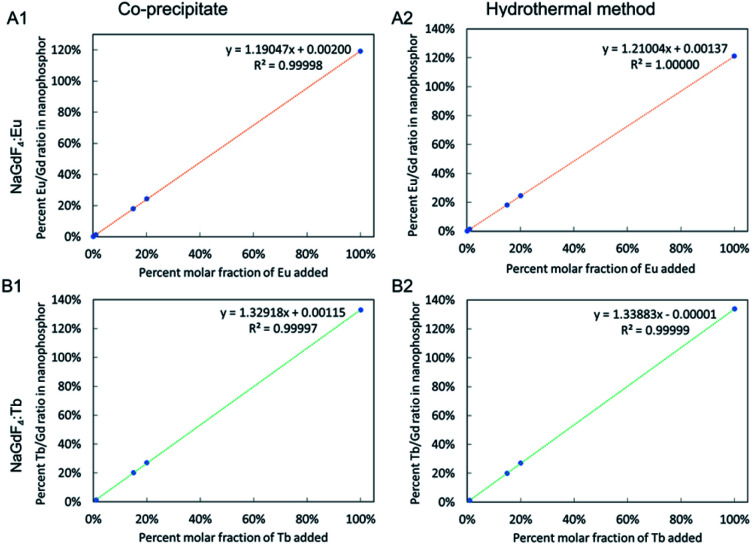
Metal composition of nanophosphors calculated using ICP-OES measurements. The graph of percent metal ratio of nanophosphor *vs.* percent molar fraction of dopant added of NaGdF_4_:Eu synthesized by (A1) co-precipitate and (A2) hydrothermal methods and NaGdF_4_:Tb synthesized by (B1) co-precipitate and (B2) hydrothermal methods. Note, the amount of Tb in NaGdF_4_:0.1%Tb was not calculated as it is lower than the limit of detection of the instrument.

**Fig. 6:**
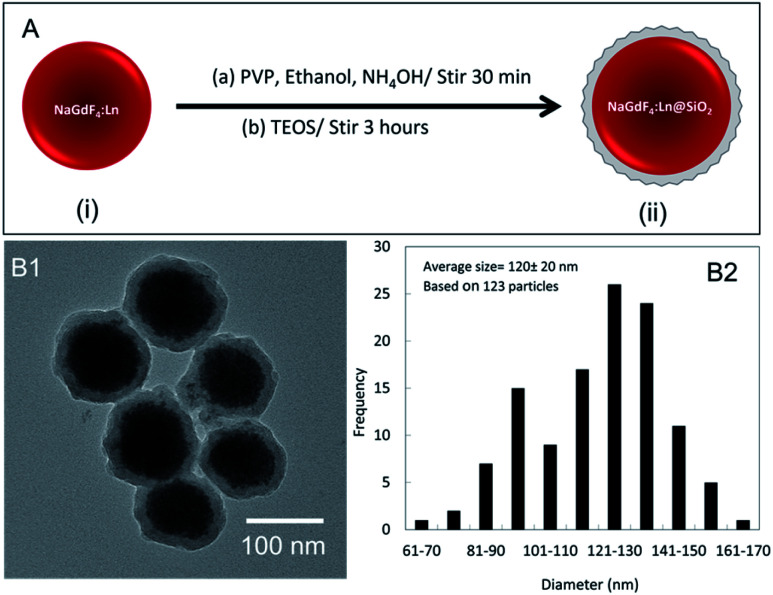
(A) Schematic illustration of nanoparticle coating with silica. (i) NaGdF_4_:Ln^3+^ nanophosphors, (ii) silica-coated NaGdF_4_:Ln^3+^ nanoparticles. (B1) TEM images of silica-coated NaGdF_4_ nanophosphors Eu-doped. (B2) Histograms of the diameter of silica-coated NaGdF_4_:Eu.

NaGdF_4_:Eu nanophosphors, synthesized at room temperature, were coated with silica before further experiments. The silica can protect nanophosphors from aggregation and facilitate functionalization for biological applications. Fig. 5A presents the NaGdF_4_ silica coating process. In electron microscopy images (Fig. 6B1) the silica coating appeared as a ∼15 nm thick shell around the nanophosphors. The average diameter of NaGdF_4_:Eu@SiO_2_ nanophosphors was about 120 nm (Fig. 5B2).

**Fig. 7:**
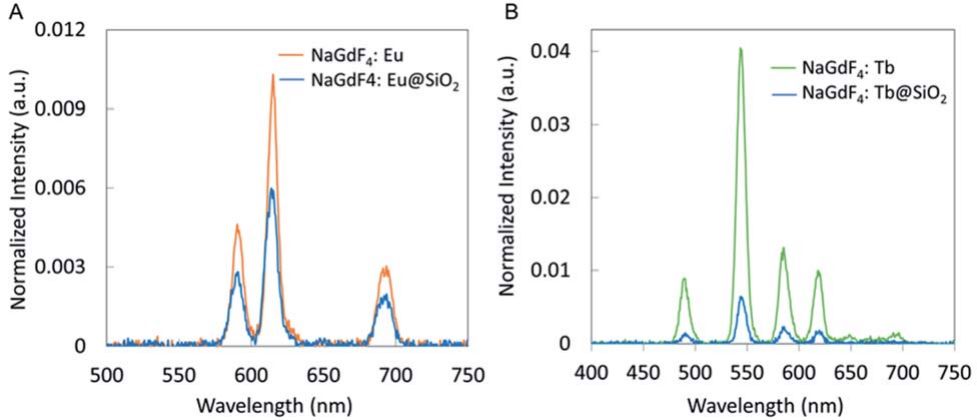
XEOL spectra of silica-coated (A) NaGdF_4_:Eu and (B) NaGdF_4_:Tb compared to NaGdF_4_:Eu and NaGdF_4_:Tb synthesized using co-precipitate method.

Both Tb-doped and Eu-doped nanophosphors showed reduced X-ray luminescence after coating with silica for the same mass of material. After coating, the NaGdF_4_:Eu nanophosphors reduced intensity by ∼42% (Fig. 7A). This is largely explained by the additional silica which increased the mass by 65% (based on particle diameter, coating thickness, and density of NaGdF_4_ core and silica shell), while not providing any additional luminescent material.

### Thermal analysis

**Fig. 8:**
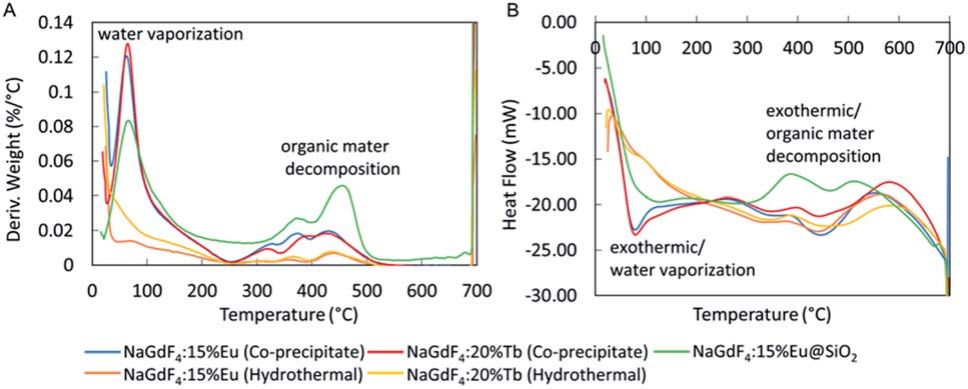
(A) TGA and (B) DSC of NaGdF_4_:Eu and NaGdF_4_:Tb nanophosphors synthesized using co-precipitate and hydrothermal methods and NaGdF_4_:Eu@SiO_2_.

The stability of the nanophosphors towards heat was studied by thermal gravimetric analysis (TGA) and differential scanning calorimetry (DSC) thermograms as in Fig. 8A and B. NaGdF_4_:Eu and NaGdF_4_:Tb synthesized using co-precipitate method showed total weight loss of 12.5% and 12.1% respectively. Their highest weight loss appeared at 62.5 °C is attributed to the loss of trapped H_2_O, and weight loss above 250 °C may cause by the decomposition of citrate. The nanophosphors synthesized using the hydrothermal method indicated 3.10% (NaGdF_4_:Eu) and 5.17% (NaGdF_4_:Tb) total weight loss. There is no significant weight loss caused by vaporization of trapped H_2_O. Also, the weight loss above 250 °C is less intense than the weight loss of co-precipitate method. Silica coated-NaGdF_4_:Eu has total weight loss of 17.4%. Its weight loss at 65.5 °C is attributed to the loss of trapped H_2_O and ethanol. The peaks above 250 °C are more intense than for uncoated nanophosphors and likely caused by decomposition of citrate and PVP organic materials (ESI Fig. S4B and C†). Further, a DSC thermogram shows that vaporization and decomposition are both exothermic processes. The TGA thermogram shows that nanophosphors synthesized by the hydrothermal method contain less trapped water and organic materials, which may result in high X-ray luminescence intensity.

### Annealing NaGdF_4_:Eu nanophosphors

**Fig. 9:**
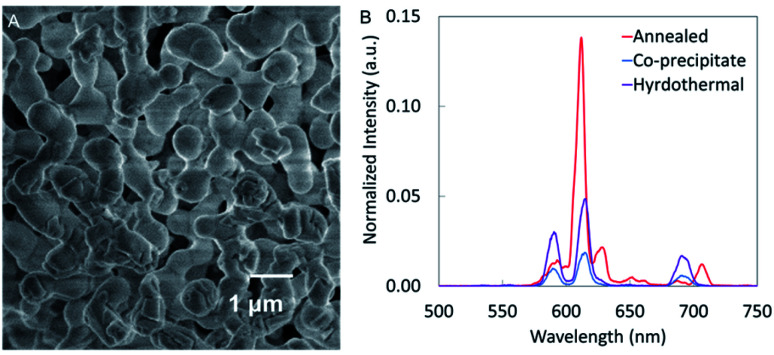
(A) SEM image and (B) XEOL of NaGdF_4_:Eu annealed at 1000 °C for 6 h compared to NaGdF_4_:Eu nanophosphors synthesized using co-precipitate and hydrothermal methods.

Nanophosphors synthesized using the co-precipitate method and coated with silica have relatively low luminescence intensity which hinders most *in vivo* applications such as deep tissue imaging and implantable biosensors. High temperature annealing could enhance luminescence by removing crystal defects, increasing crystal domain size and removing trapped water and carbon dioxide.^23,24^ Unfortunately, when we annealed our nanophosphors at temperatures above 700 °C, we found that the particles aggregated. For example, Fig. 9A shows that after thermal annealing at 1000 °C, the NaGdF_4_:Eu nanophosphors aggregated and fused making a structure too large for many biological applications. However, the luminescence intensity was increased by a factor of ∼7 compared to nanophosphors synthesized by the co-precipitate method.

**Fig. 10:**
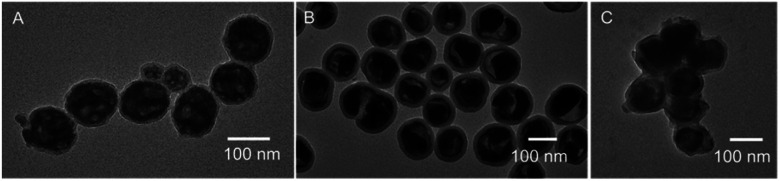
TEM images of NaGdF_4_:Eu@SiO_2_ nanophosphors annealed at (A) 400 °C, (B) 600 °C (C) 1000 °C.

Silica coating can act as a protective layer, preventing particles from aggregating and fusing during the annealing process.^24^ Annealed nanophosphors at 400 °C (Fig. 10A) showed separate particles with a thin silica coating. Annealing at 600 °C (Fig. 10B) showed single and separate nanophosphors with a silica shell, and nanophosphor core, but the core compressed, and some void space was apparent. Annealing at 1000 °C (Fig. 10C) caused the nanophosphors to aggregate, although not to the extent of forming the large porous structures observed without coating (Fig. 7C).

**Fig. 11:**
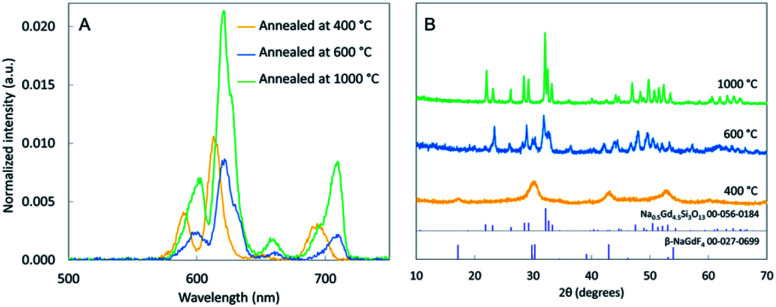
(A) XEOL of NaGdF_4_:Eu@SiO_2_ annealed at 400 °C, 600 °C and 1000 °C for 6 h. (B) Powder XRD pattern of annealed NaGdF_4_:Eu@SiO_2_ at 600 °C and 1000 °C compared to PDF cards 00-056-0184 and 00-027-0699 biotin functionalized NaGdF_4_:Eu@SiO_2_.

Annealing at 400 °C and 1000 °C increased the luminescence intensity by a factor of ∼1.5 and ∼3 compared to unannealed silica-coated nanophosphors. Among the three annealing temperatures, 1000 °C showed the highest intensity (Fig. 11A). We also observed that XEOL spectrum red-shifted at high temperature (600 °C and 1000 °C). This spectral shift could potentially be useful for generating nanophosphors with distinguishable spectra for multi-modal imaging, and likely arises from changes in composition and crystal field. (XEOL graphs of synthesized, annealed and silica coated NPs are included in ESI Fig. S3† to compare). Indeed, powder-XRD data confirmed the formation of sodium gadolinium silicate compound by reacting with the silica layer: XRD spectra display the presence of NaGd_9_Si_6_O_26_:Eu (Fig. 11B) indexed to the standard data (JCPDS 00-056-0184). Additionally, we observed that the 1000 °C annealed samples have sharper diffraction peaks indicating larger crystal domains, which likely reduces quenching from the surface and defects.^10^ Also, at 1000 °C, the nanophosphors have started to aggregate. On balance, the 1000 °C annealing appeared to give the best performance, albeit with a spectral shift. XEOL graphs of NaGdF_4_:15%Eu annealed at 150, 400 and 1000 °C for 12 h are included in ESI S5.†

### Biotin functionalized NaGdF_4_:Eu@SiO_2_

**Fig. 12:**
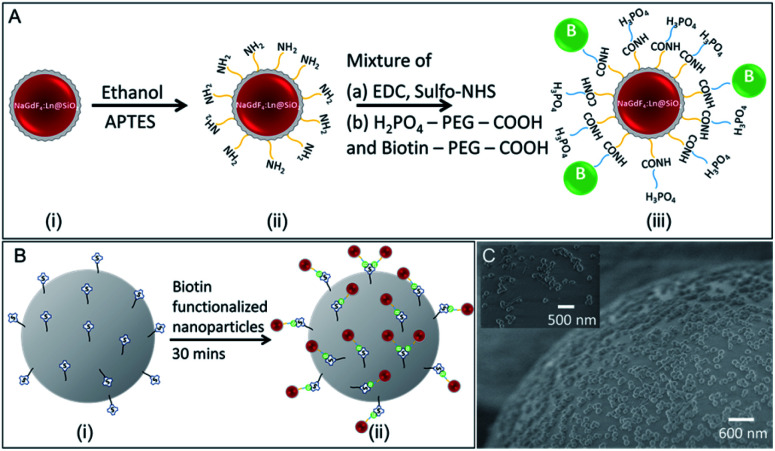
Schematic illustration of (A) silica coated nanophosphors functionalized with biotin (i) silica-coated NaGdF_4_:Eu nanophosphors, (ii) amine-functionalized nanophosphors, (iii) biotin-functionalized nanophosphors. Biotin is connected to amine-functionalized nanophosphors *via* sulfo-NHS groups, (B) biotin functionalized nanophosphors attach to streptavidin coated silica microbubbles. (i) streptavidin-coated silica microbubbles, (ii) biotin-functionalized nanophosphors attached to streptavidin-coated silica microbubbles. (C) SEM image showing biotin-functionalized nanophosphors attached to streptavidin-coated microbubbles.

Functionalizing rare-earth doped nanophosphors are important in *in vivo* labelling and imaging, biological assays and sensor applications with specific targets such as proteins and DNA.^12,32,33^ We functionalized NaGdF_4_:Eu@SiO_2_ nanophosphors (not annealed) with a mixture of PEG-phosphate and biotin-PEG. The PEG-phosphate was chosen to improve good dispersion of surface-modified nanophosphors in water at physiological pH and high ionic strength (PBS buffer). The biotin-functionalization was demonstrated by attachment to streptavidin-functionalized buoyant silica microbubbles (Fig. 12).

### X-ray excited optical luminescence spectroscopy and imaging of capillaries filled with NaGdF_4_:Eu through tissue

XEOL spectroscopy and imaging were performed to demonstrate the ability to excite the nanophosphors through tissue and show that the luminescence could be generated in specific regions using a focused X-ray source. We chose 5 mm of chicken breast because 5 mm is sufficient for many applications in a mouse model where the “radius” is <1 cm, and we used chicken breast because it is a common tissue material that scatters light. Optical scattering in chicken breast is highly anisotropic and has larger transport mean free path (the length over which the direction of photon propagation is randomized) than scattering mean free path.^34^

**Fig. 13:**
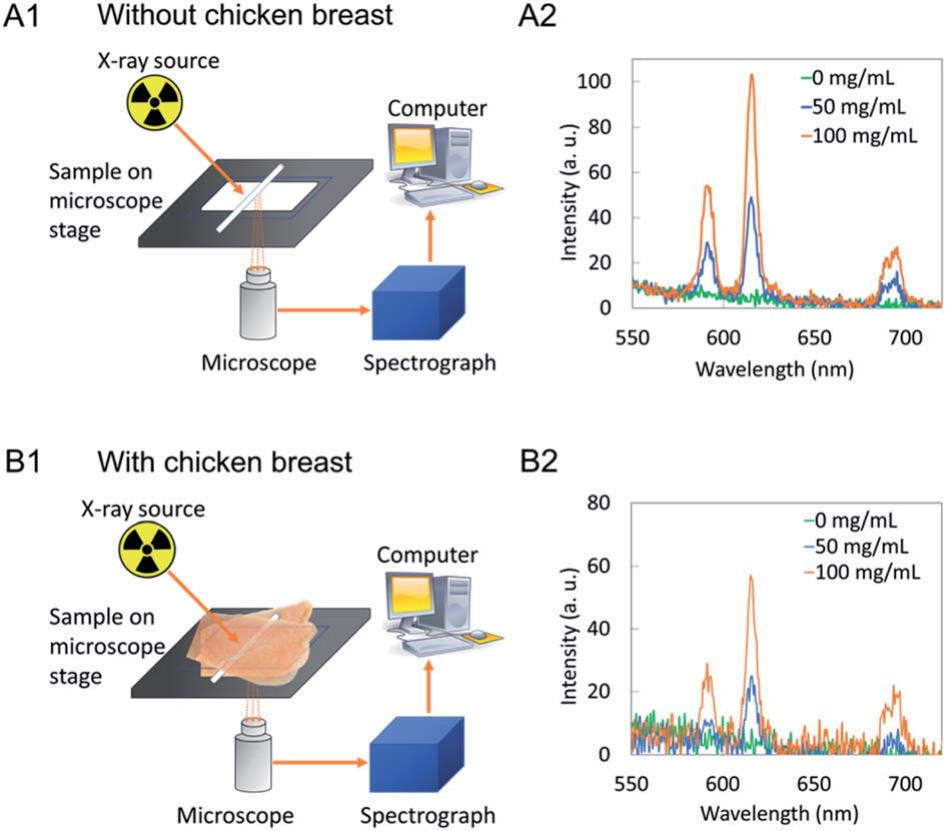
Schematic illustration of measuring XEOL of NaGdF_4_:Eu filled in capillaries (A1) without tissue (B1) sandwiched with two 5 mm porcine tissues. XEOL of NaGdF_4_:Eu filled in capillaries (A2) without tissue (B2) sandwiched with two 5 mm porcine tissues. Exposure time was 10 s without tissue and 60 s through tissue.

XEOL spectroscopy of colloidal NaGdF_4_:Eu nanophosphors in capillaries were measured with and without being sandwiched between two 4 mm porcine tissue. Fig. 13A1 and B1 show a schematic illustration of the experimental setup. The glass slide carrying capillaries (1 mm diameter) filled with a nanoparticle solution (0, 50, 100 mg mL^−1^) is placed on the microscope stage. The light generated in X-ray irradiated nanophosphors was collected through a microscope lens and send to a spectrograph. Spectra generated without tissue (Fig. 13A2) shows intensity higher than capillaries sandwiched between porcine tissues (Fig. 11B2). Also, as the concentration of nanophosphors increase in the capillary intensity increases in both scenarios.

**Fig. 14:**
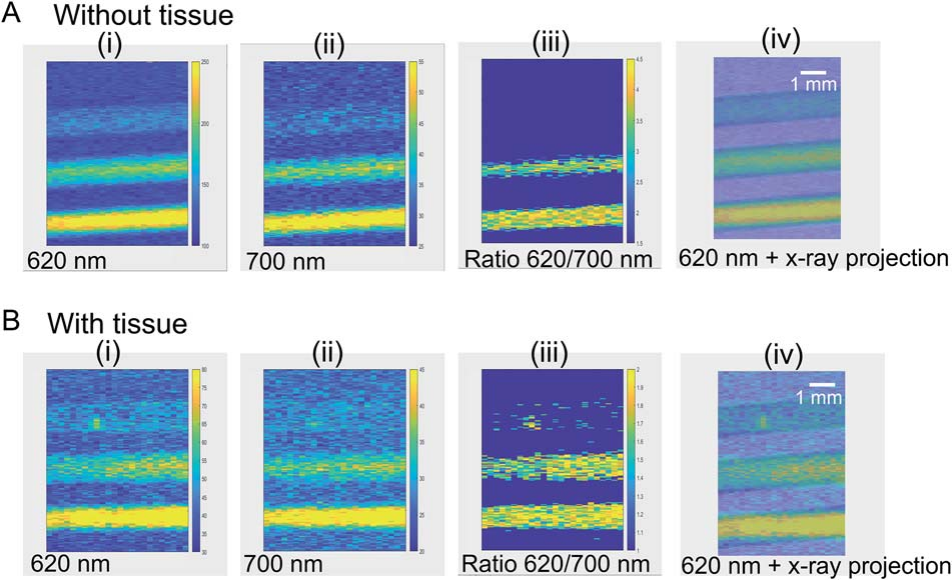
XELCI images of NaGdF_4_:Eu dispersion in capillaries (A) XELCI images without tissue (i) image of 620 nm intensity (counts) *vs.* position. (ii) Image of 700 nm intensity (counts) *vs.* position (iii) XELCI ratio image (intensity ratio of 620 nm and 700 nm). (B) XELCI images of the same capillaries sandwiched between two 5 mm porcine tissue slices (i) 620 nm image (ii) 700 nm image (iii) XELCI ratio image. (iv) Superimposed 620 nm image and X-ray transmittance images. Current was 200 μA without tissue and 600 μA through tissue. 1 mm scale bar is same for all images.

To demonstrate focused X-ray excited light generation and collection through tissue, we loaded 1 mm diameter glass capillaries with NaGdF_4_:Eu nanophosphor dispersions, and placed the capillaries beneath 5 mm porcine tissue for imaging (Fig. 14). Three capillaries were used, with NaGdF_4_ concentrations of 0, 50 and 100 mg mL^−1^, respectively. Our 0.75′′ diameter collection optics and coupled acrylic light guide collected light from a large field of view (∼1′′), but the focused X-ray beam irradiated only a small region (∼250 μm spot size). We found a relatively sharp image showing luminescence only from the filled region of the capillaries, and excellent agreement with the X-ray transmission image. The capillary with DI water (0 mg mL^−1^) produced dim luminescence, as glass displays a weak X-ray luminescence signal^35^ with a broad spectrum roughly peaking at 430 nm (ESI Fig. S6†). The signal at 50 mg mL^−1^ and 100 mg mL^−1^ are clearly much larger and increase proportionally to concentration. Importantly, although the signal through tissue was 9.6 times weaker than with no tissue (mainly due to optical attenuation and partly from X-ray attenuation), the observed spatial resolution was very similar demonstrating local radioluminescence excitation through tissue.

### MR imaging of NaGdF_4_:Eu and Tb

**Fig. 15:**
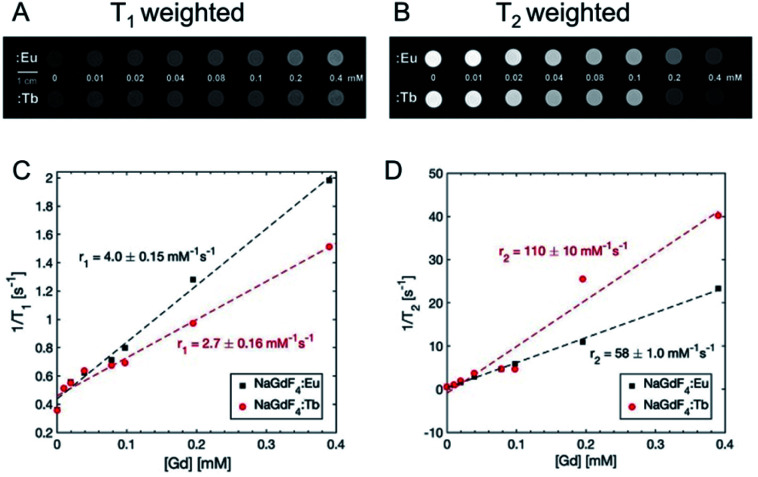
Magnetic resonance images (MRI) of NaGdF_4_:Eu and NaGdF_4_:Tb nanoparticle suspensions in water. (A) *T*_1_ weighted and (B) *T*_2_ weighted images with various nanoparticle concentrations. 0 mM = DI water. 1 cm scale bar is for both *T*_1_ and *T*_2_ weighted images. (C) Longitudinal relaxation rate *vs.* concentration for nanoparticle suspensions. (D) Transverse relaxation rate *vs.* concentration. Dashed lines are linear fits to data.

NaGdF_4_:Eu and Tb may also potentially be *T*_1_ and *T*_2_ weighted MRI contrast agents as previously reported.^36,37^ To demonstrate this, varying concentrations of NaGdF_4_:Eu and Tb were prepared in DI water and imaged using a 3T Siemens MAGNATOM Prisma MRI (Fig. 15A and B). As expected, *T*_1_ weighted images became brighter, and *T*_2_ weighted images became darker as [Gd] increased up to 0.4 mM. Fig. 13C and D show longitudinal and transverse ^1^H relaxation rates, *r*_1_ and *r*_2_, measured at 20 °C plotted against concentration of the nanoparticle suspensions. Relaxivities were calculated by linear regression of this data. The longitudinal relaxivities for NaGdF_4_:Eu and NaGdF_4_:Tb were 4.0 ± 0.15 and 2.7 ± 0.16 mM^−1^ s^−1^, and the transverse relaxivities were 58 ± 1.0 and 110 ± 10 mM^−1^ s^−1^, respectively. The observed differences between Tb and Eu doped particles, especially at high concentrations, are likely from variation in sample preparation since Tb and Eu doping is at a low concentration compared to the Gd in the host crystal. The values are in the range of prior literature and the relaxivities provide clear contrast.^36^ The reported relaxivity values 0.27 (minimum *r*_1_) and 160 (maximum *r*_2_) mM^−1^ s^−1^ depends on field strength,^36,38^ size and morphology^36,38^ and coating.^39^ MRI imaging would allow for deep tissue imaging and would be complementary to X-ray luminescence imaging and stimulation, allowing for non-invasive localization of nanophosphors *in vivo*.

## Experimental

### NaGdF_4_:Eu and NaGdF_4_:Tb nanoparticle synthesis

We synthesized particles using either the co-precipitation or hydrothermal synthesis methods.

#### Co-precipitate synthesis method

NaGdF_4_:Eu and Tb nanophosphors were synthesized by the citrate method with slight modifications.^14,40^ Sodium citrate (12 mL of 0.2 M) was added to a clear aqueous solution containing 4 mL of GdNO_3_ (0.2 M) and TbNO_3_/EuNO_3_ (*X* mol%) and stirred vigorously for 30 min at room temperature. Then, 16 mL of sodium fluoride (1 M) solution was combined with the above mixture and stirred vigorously for another 2 hours which result in a white solution. The synthesized nanophosphors were centrifuged and washed three times before further experiments. (TbNO_3_/EuNO_3_*X* mol% = 0.1%, 1%, 15% 20%, 100% of Gd^3+^ moles).

#### Hydrothermal synthesis method

NaGdF_4_:Eu and Tb nanophosphors were synthesized by the citrate method with slight modifications.^14,15,40^ Sodium citrate (12 mL of 0.2 M) was added to a clear aqueous solution containing 4 mL of GdNO_3_ (0.2 M) and TbNO_3_/EuNO_3_ (*X* mol%) and stirred for 30 min. Then, 16 mL of sodium fluoride (1 M) solution was combined with the above mixture and stirred vigorously for another 15 minutes which result in a white solution. This solution was transferred to a Teflon-lined autoclave and heated at 180 °C for 6 hours. The synthesized nanophosphors were centrifuged and washed three times before further experiments. The synthesized nanophosphors were stored in either DI water or 0.1% citrate solution. (TbNO_3_/EuNO_3_*X* mol% = 0.1%, 1%, 15% 20%, 100% of Gd^3+^ moles).

### Silica coating NaGdF_4_:Eu and Tb nanoparticles

Synthesized nanophosphors were coated with silica using previously described methods^41–43^ with slight modifications (certain reagents were scaled up). Eu doped and Tb doped NaGdF_4_ nanoparticles prepared at room temperature were resuspended in 8 mL of water and combined with 200 mL ethanol solution containing PVP (1.2 g) and ammonium hydroxide (6 mL). Then, TEOS (160 μL) was added after the solution was stirred for 20 min. The particles were aged another 3 hours before centrifuged and washed using DI water.

### Annealing NaGdF_4_:Eu nanoparticles

NaGdF_4_:Eu nanoparticles prepared using the co-precipitate method were dried at 80 °C to form a white powder. It was transferred to a crucible and heated (10 °C min^−1^) at 1000 °C for 6 hours in a muffle furnace which results in a solid, aggregated structure. After cooling down to room temperature, it was crushed into a fine powder using mortar and pestle.

NaGdF_4_:Eu@SiO_2_ nanophosphors were dried at 80 °C to form a white powder and divide into three portions and transferred to crucibles. The samples were heated at 400 °C, 600 °C and 1000 °C for 6 hours to anneal them.

### Functionalization of NaGdF_4_:Eu@SiO_2_ with biotin

NaGdF_4_:Eu@SiO_2_ nanophosphors were resuspended in ethanol (100 mL) with APTES (300 μL) and stirred for 3.5 h. The amine-functionalized nanophosphors were collected and washed three times using DI water.

98 mL of MES buffer (0.1 M, pH 6.0) was taken in a 500 mL round bottom flask. ∼100 mg of water-soluble carbodiimide (EDC) and sulfo-NHS was added to it and stirred for 15 minutes at room temperature. H_2_PO_4_–PEG–COOH (5000 Da) (500 μL, 10 mg mL^−1^) and biotin–PEG–COOH (1000 Da) (250 μL, 10 mg mL^−1^) was added to the above solution and stirred for 1 hour at room temperature to activate COOH groups. Then, the solution pH was adjusted to pH 7.4 using PBS buffer. Previously prepared NH_2_ functionalized NaGdF_4_:Eu@SiO_2_ nanoparticles were added to the mixture and it could react for 12 h at room temperature with continuous stirring. Lastly, biotin-conjugated NPs were washed with PBS three times and stored in DI water.

Biotin functionalized aqueous nanophosphors suspension (10 mg mL^−1^, 20 μL) was transferred into a centrifuge tube contained PBS (10×, 380 μL) solution. Streptavidin-coated microbubbles (100 μL) were mixed with the previous solution and vortexed to allow particle adhesion on the surface of the microbubble. After waiting a 10–15 minutes, suspended microbubbles were pipetted out and washed three times with DI water.^44,45^

### Imaging capillaries filled with NaGdF_4_:Eu through tissue

A series of concentration of NaGdF_4_:Eu (100, 50 and 0 mg mL^−1^) was prepared and filled in 1 mm (inner diameter) capillaries.

X-ray excited optical luminescence of all the samples in capillaries were measured by irradiating with a Mini-X Ag-target X-ray source at 40 kV and 99 μA. Spectral data of capillaries were obtained at 10 s exposure time and capillaries sandwiched in between 4 mm porcine tissue were obtained at 60 s exposure time.

X-ray excited luminescence chemical imaging (XELCI) of these capillaries was done without tissue and with 5 mm porcine tissue. The capillaries were scanned with 250 μm step size and 1 mm s^−1^ for a high-resolution scan. For imaging without tissue, the X-ray source was set to 50 kV and 200 μA; 50 kV and 600 μA was used for imaging with porcine tissue. Data were analysed and plotted using custom MATLAB scripts.

### MR imaging of NaGdF_4_:Eu and Tb

Solutions of NaGdF_4_:Eu and Tb nanophosphors at a series of concentrations from 0 to 0.4 mM were prepared by dissolving in DI water. MR imaging and relaxometry was done on a 3T Siemens MAGNATOM Prisma MRI instrument (Siemens Healthineers, Erlangen, Germany). *T*_1_-weighted images were acquired using a 2D spin echo sequence with repetition time (TR) 25 ms and echo time (TE) 5.9 ms. *T*_2_-weighted images were acquired using a turbo spin echo sequence with TR = 3200 ms and TE = 29 ms. *T*_1_ was measured with an inversion recovery experiment using a spin echo pulse sequence. Inversion times (TI) were 25, 50, 400, 1100, and 2500 ms. To calculate *T*_1_, the model from ref. 46 was fit to MR signal magnitude *vs.* TI using the qMRLab package for MATLAB.^47^*T*_2_ was measured with a multi-echo spin echo sequence using 32 TEs ranging from 15 to 960 ms. To calculate *T*_2_, a monoexponential model 
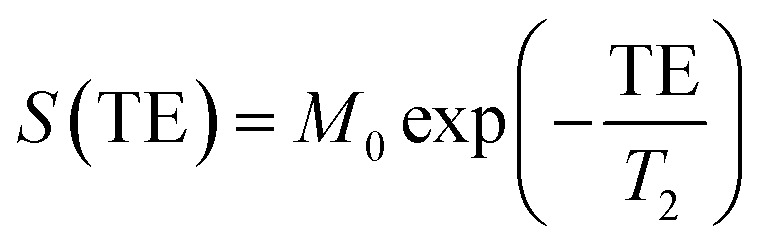
 was fit to MR signal magnitude *vs.* TE. Relaxivities were calculated by linear regression of *R*_1_ = 1/*T*_1_, *R*_1_ = 1/*T*_2_*vs.* nanoparticle concentration.

### Transmission electron microscopy

The synthesized, silica-coated and annealed nanoparticles were deposited on a formvar/carbon-coated copper grids from a water solution and dried before taking the transmission electron micrographs from Hitachi HT7800 operating at 20–120 keV and 8 μA.

### Powder X-ray diffraction (XRD)

The phases of the NaGdF_4_:Eu powders, nanoparticles, and annealed nanoparticles were characterized by Rigaku Ultima IV powder diffractometer, using Cu K_α_ radiation. Powdered samples were spread on a low background glass slide and data are collected in 0.02° increments at a rate of 0.5–1° per minute from 10° to 70° at room temperature. Annealed particles were measured at 0.2–0.5° per minute.

### X-ray excited optical luminescence (XEOL) spectroscopy

X-ray luminescence of all the nanoparticles was measured by irradiating with an X-ray beam. A 96 well plate containing dried and powdered nanoparticles was places on the stage of an inverted microscope and irradiated with an X-ray beam generated using a Mini-X (Ag) X-ray source (Amptek, Bedford, MA) set at 40 kV and 99 μA. The emission of nanoparticles was collected by 5× objective and focused to a spectrograph (DNS 300, DeltaNu, Laramie, WY, USA), equipped with a cooled CCD camera (iDUS-420BV, Andor, South Windsor, CT, United States).

### ICP-OES metal analysis

The percentage of metals (Gd, Eu and Tb) in the final systems were measured using iCAP 7200 MSC ICP-OES analyser. Nanophosphors were dissolved in 2% HNO_3_ to prepare nitrates of the metals. The standard metal solutions were prepared from 0.1 to 100 ppm range. The samples and the standards were injected to ICP-OES and quantified the amount of metal using instrument software (Qtegra ISDS software). The wavelengths of the corresponding spectrometric lines that used for the analysis were Gd: 335 nm, Eu: 381 nm, Tb: 350 nm. The metal percentages (Eu/Gd and Tb/Gd) from the dissolved samples were calculated using standard curves.

### Thermal analysis: TGA/DSC

TGA and DSC measurements were carried out on a SDT Q600 V20.9 Build 20 thermal gravimetric by TA instruments. Nanophosphor samples in alumina crucibles were heated up to 700 °C at a rate of 20 °C minute^−1^ under N_2_ gas flow. At 700 °C samples were kept under isothermal conditions and flushed with air. TGA and DSC measurements were collected simultaneously.

## Conclusions

In summary, we described two methods to synthesis Eu- and Tb doped NaGdF_4_ X-ray luminescence nanophosphors. The co-precipitation method is a simple method that synthesized spherical-shaped nanoparticles at room temperature. The hydrothermal method generated irregular particles that yielded higher X-ray excited luminescence intensity. Our investigation on annealing nanoparticles revealed that nanoparticles without a silica coating resulted in high luminescence intensity yet aggregated particles. The silica coating act as a protection layer to prevent aggregation and increase X-ray luminescence during the annealing process. However, at high temperature, it transformed NaGdF_4_:Eu into sodium gadolinium silicate which also shows X-ray luminescence. Then, we functionalized NaGdF_4_:Eu@SiO_2_ nanophosphors with biotin and confirmed attaching to streptavidin *in vitro*. Importantly, we selectively excited scintillator nanophosphors using a focused X-ray source to generate light and collect them through the tissue to generate XELCI images and XEOL was measured through tissue. We also showed that the particles could serve as MRI contrast agents. Future work will investigate the spectral shift by changing host materials for multi-analyte imaging through tissue.

## Author contributions

J. N. Anker conceptualisation, funding acquisition, supervision, writing-review and editing; M. Ranasinghe data curation, investigation, methodology, validation, writing – original draft preparation; M. Arifuzzaman, A. C. Rajamathrilage, W. R. Willoughby, and A. Dickey investigation, validation; Colin McMillen data curation; J. W. Kolis supervision, M. Bolding supervision.

## Conflicts of interest

There are no conflicts to declare.

## Supplementary Material

RA-011-D1RA05451A-s001
