# Supplementary material for: X-ray excited luminescence spectroscopy and imaging with NaGdF4:Eu and Tb
Source: RSC Adv. 2021 Sep 24;11(50):31717–26. doi: 10.1039/d1ra05451a (PMC9041542; doi:10.1039/d1ra05451a)
Supplement: RA-011-D1RA05451A-s001 [file RA-011-D1RA05451A-s001.pdf]

Electronic Supplementary Material (ESI) for RSC Advances.
This journal is © The Royal Society of Chemistry 2021

Supporting Information: X-Ray Excited Luminescence Spectroscopy and Imaging with $NaGdF_4$:Eu and Tb

Meenakshi Ranasinghe[a], Md. Arifuzzaman[a], Apeksha C. Rajamanthrilage[a], W. R. Willoughby[b] Ashley Dickey[a], Colin McMillen[a], Joseph W. Kolis[a], Mark Bolding[b], Jeffrey N. Anker*[a]

[a]Department of Chemistry, Clemson University, Clemson, SC; and Center for Optical Materials Engineering and Technology (COMSET)

[a]Department of Radiology, University of Alabama at Birmingham School of Medicine, Birmingham, AL;

E-Mail: mranasi@g.clemson.edu; marifuz@g.clemson.edu; apekshr@g.clemson.edu; dickey@g.clemson.edu; cmcmill@clemson.edu; kjoseph@clemson.edu; mbolding@uabmc.edu; janker@clemson.edu

Figure		Page
S1	TEM images of $NaGdF_4$:Eu and XRD patterns of Eu- and Tb-doped $NaGdF_4$ synthesized using the co-precipitate method.	2
S2	TEM images of $NaGdF_4$:Eu and XRD patterns of Eu- and Tb-doped $NaGdF_4$ synthesized using the hydrothermal method.	2
S3	X-ray excited optical luminescence (XEOL) spectra of synthesized, silica coated, and silica coated annealed Eu-dopped $NaGdF_4$ NPs.	3
S4	TGA thermograms of synthesized nanophosphors, sodium citrate and PVP.	3
S5	XEOL spectra of $NaGdF_4$:Eu@SiO_2 unannealed and annealed at 150, 400, 600 °C for 12 hours.	4
S6	Fullscale XEOL spectra of $NaGdF_4$: Eu in capillaries A) without tissue B) sandwiched between 5 mm slices of porcine tissue.	4

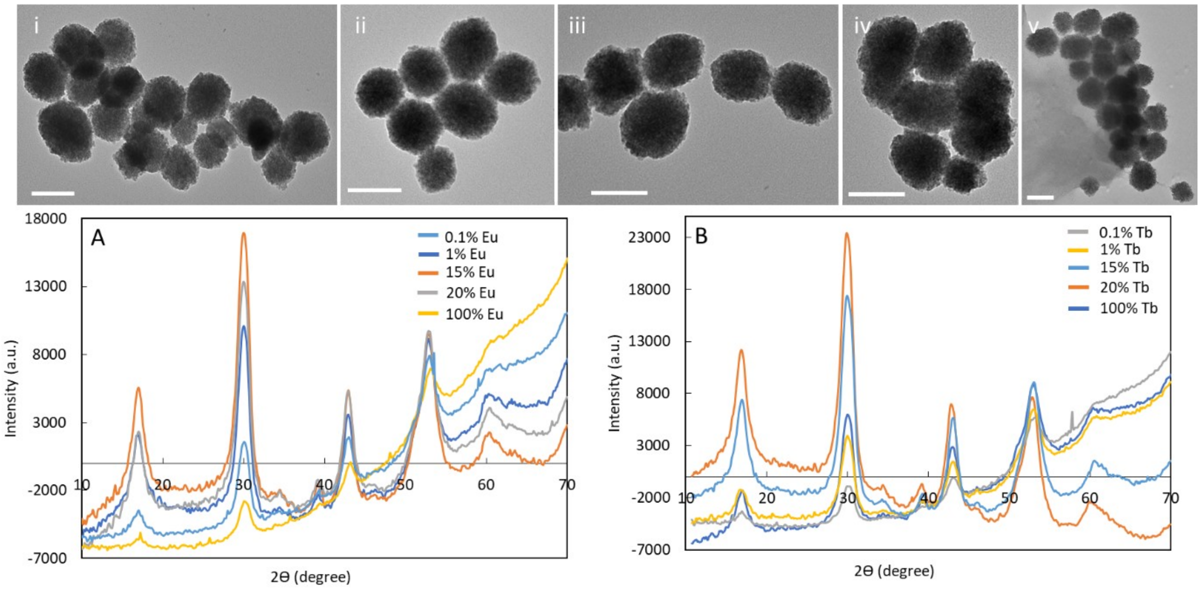

Figure S1. TEM images of $NaGdF_4$ doped with (i) 0.1%, (ii) 1%,(iii) 15%,(iv) 20%,(v) 100% Eu synthesized using the co-precipiate method. Scale bar is equal to 100 nm. XRD patterns of (A) Eu- and (B) Tb-dopped $NaGdF_4$ (Gd: dopant molar ratio= 1: 0.1, 1, 15, 20, 100%). XRD data were collected using single-crystal XRD as an alternative of powder XRD. PDF card is 27-0699.

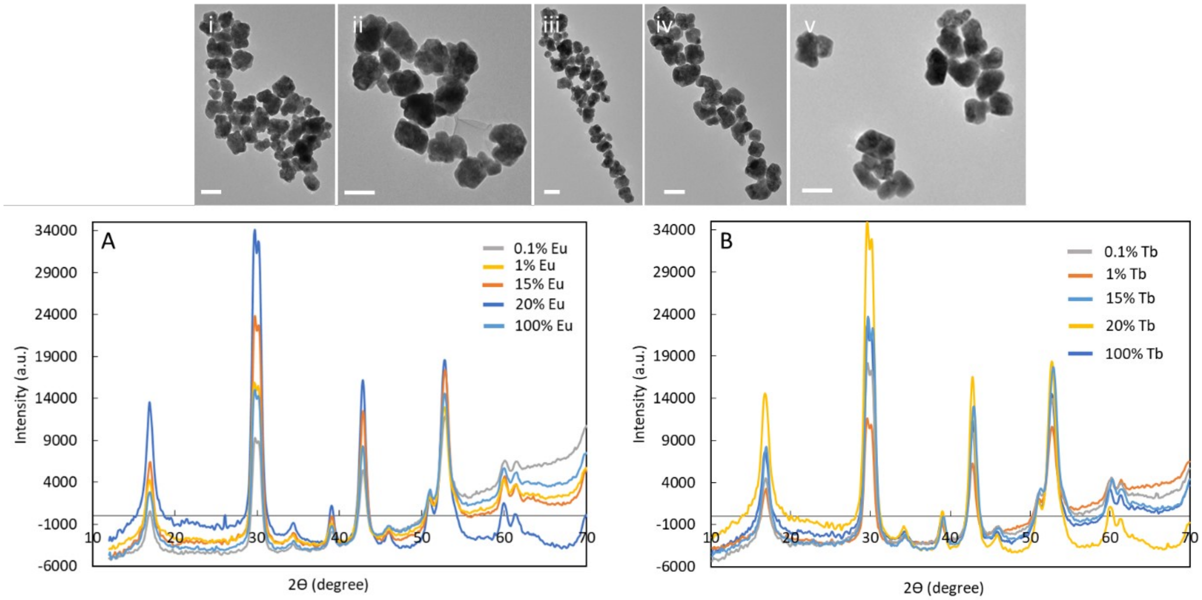

Figure S2. TEM images of $NaGdF_4$ doped with (i) 0.1%, (ii) 1%,(iii) 15%,(iv) 20%,(v) 100% Eu synthesized using the hydrothermal method. Scale bar is equal to 100 nm. XRD patterns of (A) Eu- and (B) Tb-dopped $NaGdF_4$ (Gd: dopant molar ratio= 1: 0.1, 1, 15, 20, 100%). XRD data were collected using single-crystal XRD as an alternative of powder XRD. PDF card is 27-0699

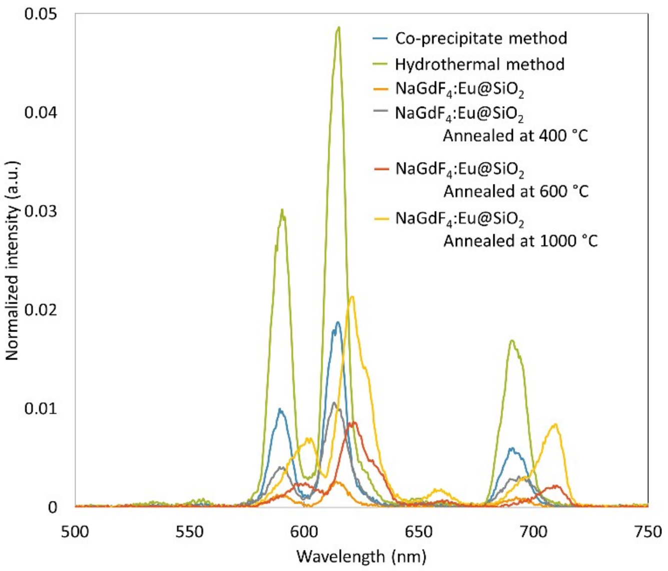

Figure S3. X-ray excited optical luminescence (XEOL) spectra of bare synthesized, silica coated, and silica coated annealed Eu-dopped $NaGdF_4$ NPs.

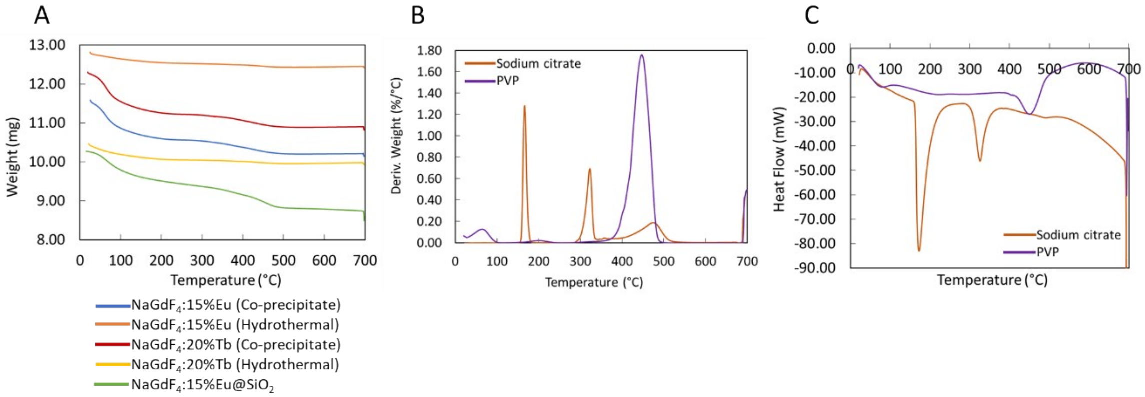

Figure S4. TGA thermograms showing (A) weight loss of synthesized nanophosphors, (B) derivative of weight loss (C) DSC of sodium citrate and PVP.

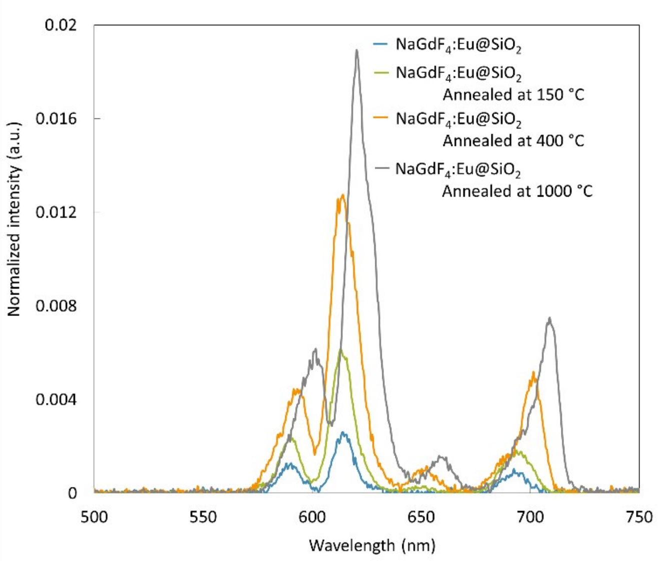

Figure S5. XEOL spectra of $NaGdF_4$:Eu@SiO_2 unannealed and annealed at 150, 400, 1000 °C for 12 hours.

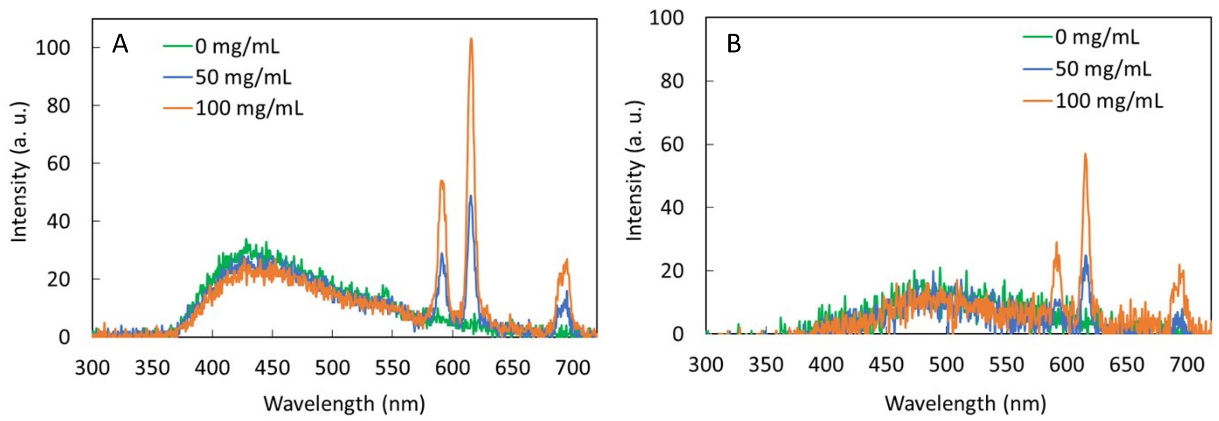

Figure S6. Full-scale XEOL of $NaGdF_4$: Eu in capillaries A) without tissue B) sandwiched between 5 mm slices of porcine tissue.
